# Perioperative management of pancreaticoduodenectomy for pancreatic ductal adenocarcinoma in a patient with moyamoya disease: a rare case report

**DOI:** 10.3389/fonc.2026.1912208

**Published:** 2026-08-25

**Authors:** Shuhao Bi, Zheng Wang, Hongyu Zhuang, Bo Hu, Lei Liu, Junke Wang, Hongxu Chen, Xiaojiang Ning

**Affiliations:** 1 Department of Trauma Surgery, The People’s Hospital of Yubei District of Chongqing (The First Affiliated Hospital of Chongqing Medical University Yubei Hospital), Chongqing, China; 2 Department of Hepatobiliary and Pancreatic Surgery, The People’s Hospital of Yubei District of Chongqing (The First Affiliated Hospital of Chongqing Medical University Yubei Hospital), Chongqing, China; 3 Department of Gastrointestinal Surgery, The People’s Hospital of Yubei District of Chongqing (The First Affiliated Hospital of Chongqing Medical University Yubei Hospital), Chongqing, China

**Keywords:** moyamoya disease, multidisciplinary team, pancreatic ductal adenocarcinoma, pancreaticoduodenectomy, perioperative care

## Abstract

**Background:**

Moyamoya disease (MMD) is a rare chronic occlusive cerebrovascular disorder associated with a high risk of perioperative ischemic or hemorrhagic stroke. Pancreatic ductal adenocarcinoma (PDAC) is a highly malignant digestive system tumor with high surgical risk. The coexistence of MMD and PDAC is extremely rare, and no consensus on standardized perioperative management protocols has been established to date. The core clinical challenge lies in balancing the goal of radical tumor resection against the prevention of potentially devastating perioperative cerebrovascular events.

**Case presentation:**

A 62-year-old Asian man with a 5-year history of asymptomatic MMD (managed with aspirin 100 mg daily) presented with painless obstructive jaundice and 5-kg unintentional weight loss. Imaging examinations demonstrated a 1.5 × 1.4 cm nodular lesion in the pancreatic head, which involved the intramural segment of the common bile duct and the duodenum. The preliminary diagnosis was pancreatic cancer. Cerebral computed tomography perfusion (CTP) showed compensatory cerebral blood volume elevation with a focal ischemic penumbra. The disease was classified as Suzuki stage III.

**Management and outcomes:**

A multidisciplinary team (MDT) formulated an individualized perioperative strategy: (1) strict hemodynamic regulation with mean arterial pressure (MAP) maintained at 80–100 mmHg, (2) optimized anesthesia with continuous regional cerebral oxygen saturation (rSO2) monitoring, and (3) staged balanced anticoagulation with low-molecular-weight heparin (LMWH) bridging. The patient underwent standard pancreaticoduodenectomy (PD). The patient had an uneventful perioperative course, with no cerebrovascular events or major surgical complications. The final pathological examination confirmed moderately differentiated PDAC (pT1cN0M0, AJCC 8th edition) with R0 resection. During postoperative follow-up, the patient exhibited no tumor recurrence, neurological deficits, or cerebrovascular events.

**Conclusion:**

For patients with MMD undergoing radical PD, MDT-guided individualized management centered on cerebral perfusion maintenance, hemodynamic stability, and staged anticoagulation may help mitigate perioperative risks and achieve favorable clinical outcomes. This case provides practical experience for clinicians confronted with similarly rare clinical conditions. Given the inherent limitations of a single-case design, its generalizability remains to be validated in larger multicenter cohorts.

## 1 Introduction

Moyamoya disease (MMD) is a rare, idiopathic, progressive occlusive cerebrovascular disease with an incidence of approximately 0.5 per 100,000 population,characterized by progressive stenosis or occlusion of the terminal internal carotid arteries bilaterally and the formation of fragile, abnormal collateral “moyamoya” vessels at the base of the brain (1). The core clinical risk of MMD arises from impaired cerebrovascular autoregulation and hemodynamic instability, which can precipitate ischemic stroke from hypoperfusion or intracranial hemorrhage from collateral vessel rupture, especially in the perioperative setting when surgical stress and circulatory perturbations are common (2). Pancreatic ductal adenocarcinoma (PDAC) is a highly malignant digestive system tumor with an extremely poor prognosis, having an incidence of approximately 4.7 per 100,000 population. Pancreaticoduodenectomy (PD) remains the only potentially curative treatment for PDAC; however, this procedure is associated with severe surgical trauma and marked perioperative hemodynamic fluctuations (3).

The coexistence of MMD and PDAC constitutes an extremely rare clinical scenario, with only a handful of case reports published worldwide. Perioperative management of patients with MMD and concurrent malignancy presents a unique dual clinical challenge: achieving oncologically radical resection while preventing life-threatening cerebrovascular events triggered by hemodynamic instability (4). To date, no published studies have systematically delineated perioperative management strategies for this high-risk patient population.

We report a rare case of asymptomatic MMD concurrent with resectable PDAC, in which the patient underwent standard PD under multidisciplinary team (MDT)-guided individualized perioperative management and achieved favorable clinical outcomes. This report aims to share our perioperative management experience from this rare case and provide a clinical reference for clinicians encountering similar scenarios.

## 2 Case presentation

A 62-year-old Asian man patient was admitted to our department with chief complaints of painless obstructive jaundice, anorexia and 5 kg weight loss within 1 month. The patient had a 5-year confirmed diagnosis of idiopathic MMD and had been regularly follow-up in the neurology department. The patient had no history of transient ischemic attack (TIA), ischemic stroke, or intracranial hemorrhage, and had never undergone cerebrovascular bypass or revascularization surgery. The patient had been receiving long-term oral low-dose aspirin (100 mg daily) for primary ischemic prevention, with strict blood pressure control (systolic blood pressure [SBP] maintained at 120–130 mmHg). The patient had no other chronic comorbidities, was a non-smoker and non-drinker, and had no family history of hereditary diseases.

Vital signs were stable (blood pressure 125/78 mmHg, heart rate 72 beats per minute [bpm]). The patient was conscious with clear orientation. Marked scleral and cutaneous jaundice was observed, with no abdominal tenderness or palpable masses. Laboratory findings revealed elevated total bilirubin (172.8 μmol/L), direct bilirubin (105.0 μmol/L), and carbohydrate antigen 19-9 (CA19-9, 170.9 U/mL). Routine blood tests, coagulation profile, renal function, and electrolyte levels were within normal limits.

**Figure 1:**
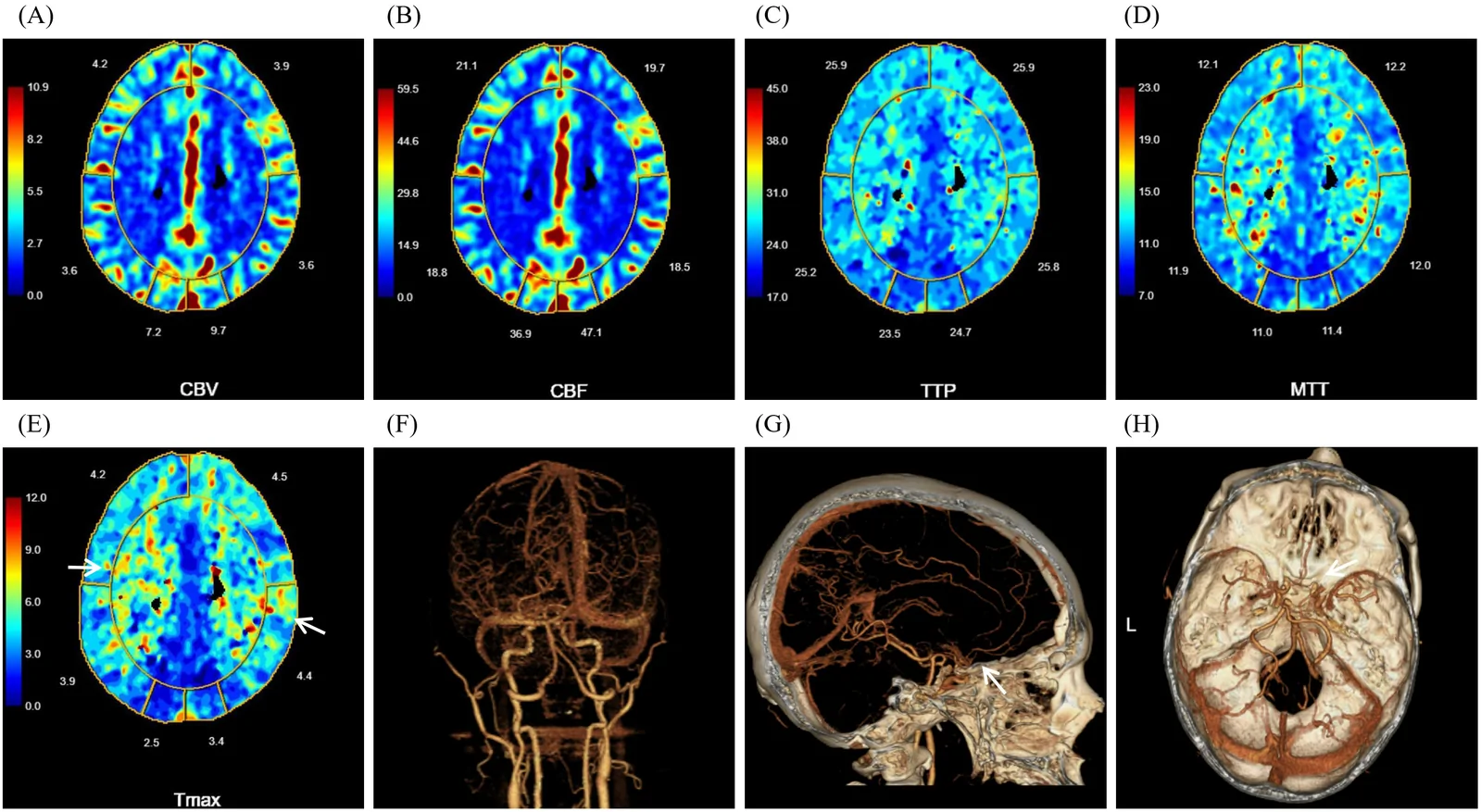
CT perfusion (CTP) and CT Angiography (CTA) images demonstrating characteristic imaging findings of MMD. **(A–E)** CBV shows compensatory elevation in the deep gray matter; CBF shows cortical hypoperfusion; MTT, TTP, and Tmax are diffusely prolonged, with a focal ischemic penumbra (indicated by yellow, orange, and red areas). **(F–H)** CTA reveals occlusion of the A1 segments of the bilateral anterior cerebral arteries and the M1 segments of the bilateral middle cerebral arteries, with surrounding “puff of smoke” collateral vessels (indicated by white arrows). The disease was classified as Suzuki stage III. (CBV, cerebral blood volume; CBF, cerebral blood flow; MTT, mean transit time; TTP, time to peak;Tmax, time to maximum; MMD, moyamoya disease).

**Figure 2:**
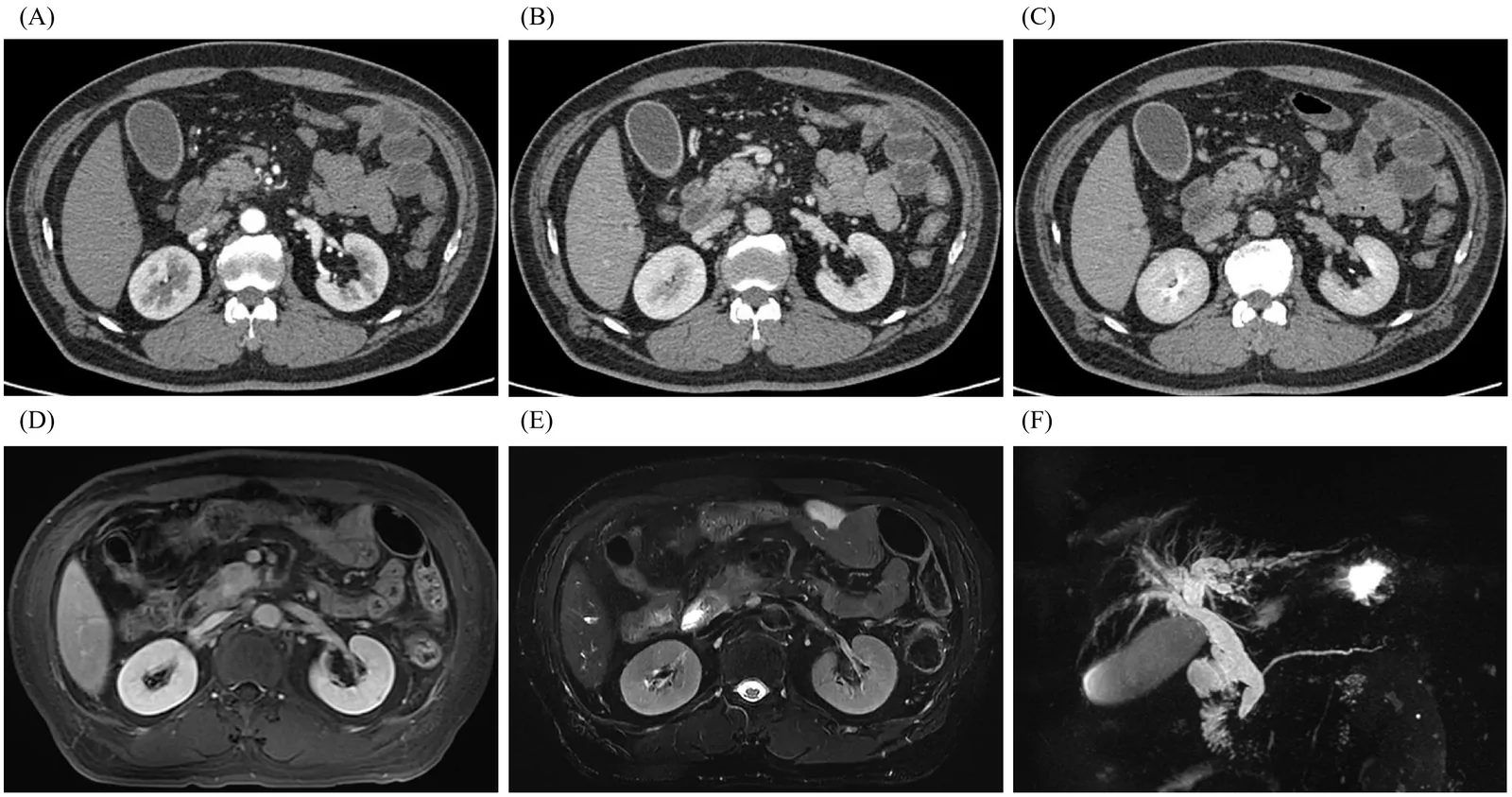
Preoperative abdominal imaging findings. **(A–C)** Contrast-enhanced CT images show an enhancing nodular lesion in the pancreatic head, which involves the intramural segment of the common bile duct. **(D, E)** Contrast-enhanced abdominal MRI images show a nodular lesion in the pancreatic head involving the intramural segment of the common bile duct. The lesion shows iso- to slight hypointensity on T1-weighted images and isointensity on T2-weighted images, with mild persistent enhancement on post-contrast sequences. **(F)** MRCP shows the classic “double duct sign”, with abrupt ductal obstruction at the level of the tumor.(CT, computed tomography; MRI, magnetic resonance imaging; MRCP, magnetic resonance cholangiopancreatography).

Cerebral blood volume (CBV) demonstrated compensatory elevation in the deep gray matter, cerebral blood flow (CBF) showed cortical hypoperfusion, and mean transit time (MTT), time to peak (TTP), and Tmax were diffusely prolonged with a focal ischemic penumbra, consistent with the typical computed tomography perfusion (CTP) features of MMD (**Figures 1A–E**). Cranial CT Angiography (CTA) reveal occlusion of the A1 segment of the bilateral anterior cerebral artery and the M1 segment of the bilateral middle cerebral artery, with surrounding “puff of smoke” collateral vessels. The disease was classified as Suzuki stage III (**Figures 1F–H**). Preoperative abdominal imaging included triphasic contrast-enhanced CT (**Figures 2A–C**), contrast-enhanced MRI (**Figures 2D, E**) and MRCP (**Figure 2F**). Imaging examinations demonstrated a 1.5 × 1.4 cm nodular lesion in the pancreatic head. This lesion involves the intramural segment of the common bile duct and the duodenum, and is associated with the classic ‘double duct sign.’ The preliminary diagnosis is pancreatic cancer (PC). No evidence of vascular invasion, regional lymphadenopathy, or distant metastases is detected.

To clarify the diagnosis and treatment plan, a multidisciplinary team (MDT) meeting was convened, involving the departments of hepatobiliary surgery, neurology, anesthesiology, cardiovascular medicine, radiology, and the intensive care unit (ICU). The core consensus was as follows: (1) Tumor Assessment: The tumor was clinically staged as resectable PC (clinical stage T1N0M0), The patient presented with significant jaundice and concurrent moyamoya disease. Surgical delay would further increase the risk of perioperative stroke. Given the clear indication for curative resection, neoadjuvant chemotherapy was not considered at this stage; (2) Neurological Assessment: The patient had a National Institutes of Health Stroke Scale (NIHSS) score of 0. Preoperative imaging categorized the patient as Suzuki stage III, suggestive of compromised cerebral perfusion reserve and elevated risk of perioperative stroke. Given the urgency of curative resection for PC, PD was performed first alongside optimized perioperative anticoagulation management. Cerebrovascular reconstruction would be considered upon reassessment once the oncological condition stabilized; (3) Management Principles: Hemodynamic Regulation: Blood pressure was strictly controlled throughout the perioperative period (maintained within ±10% of the baseline level), and mean arterial pressure (MAP) was maintained at 80–100 mmHg to avoid both hypotension-induced cerebral hypoperfusion and hypertension-related collateral vessel rupture. Anesthetic Management: Selection of anesthetic agents with minimal impact on cerebral blood flow. Maintenance of normocapnia (partial pressure of arterial carbon dioxide, PaCO_2_ 35–40 mmHg), and continuous invasive arterial blood pressure monitoring and regional cerebral oxygen saturation (rSO_2_) monitoring were performed throughout the procedure; (4) Balanced Anticoagulation: Aspirin was discontinued 7 days preoperatively and bridged with subcutaneous low-molecular-weight heparin (LMWH) 4000 IU once daily, which was stopped 24 hours before surgery. Prophylactic LMWH was to be initiated 48 hours postoperatively if no evidence of bleeding. Aspirin was to be resumed once postoperative bleeding risk had adequately subsided.

(5) Postoperative Monitoring: Transfer to ICU for at least 48 hours of continuous neurological and hemodynamic monitoring. Daily NIHSS assessments to detect early neurological deterioration; (6) Enhanced Recovery After Surgery (ERAS): Implement standardized ERAS protocol to reduce surgical stress and accelerate recovery.

The patient underwent standardized PD under general anesthesia. Anesthesia induction was performed with etomidate and propofol. Anesthesia was maintained with sevoflurane combined with continuous infusion of remifentanil to ensure stable depth of anesthesia. Intraoperatively, continuous invasive right radial arterial blood pressure monitoring, central venous pressure (CVP) monitoring, and bilateral frontal rSO_2_ monitoring were performed throughout the procedure, with rSO_2_ maintained between 60% and 80%. Blood pressure was regulated via continuous norepinephrine (0.05 μg/kg/min) infusion through a syringe pump to stabilize MAP at 80–100 mmHg. Intraoperative goal-directed fluid therapy (GDFT) was implemented to optimize fluid infusion volume. The surgery was completed by an experienced hepatopancreatobiliary surgical team, including complete tumor resection, standard regional lymphadenectomy and digestive tract reconstruction. Total operative time was 420 minutes. Intraoperative vital signs remained persistently stable without significant blood pressure fluctuations. Intraoperative blood loss was 500 mL, without allogeneic blood transfusion.

The patient was immediately transferred to the ICU for monitoring postoperatively, with continuous monitoring of invasive arterial blood pressure, CVP, heart rate, oxygen saturation, and neurological function. Norepinephrine infusion via syringe pump was continued to maintain MAP within the target range, and was gradually tapered and discontinued 24 hours postoperatively. Abdominal CT performed 48 hours postoperatively showed no evidence of intra-abdominal hemorrhage, and prophylactic anticoagulation with subcutaneous LMWH 4000 IU once daily was initiated. On postoperative day 2, the patient was alert and oriented with normal neurological function, and was transferred to the general ward. Strict blood pressure control was continued and the ERAS protocol was implemented: the patient ambulated early postoperatively and resumed oral intake on postoperative day 5. Routine postoperative treatments included anti-infective therapy, acid suppression, pancreatic enzyme secretion inhibition, hepatoprotective therapy, and nutritional support.

**Figure 3:**
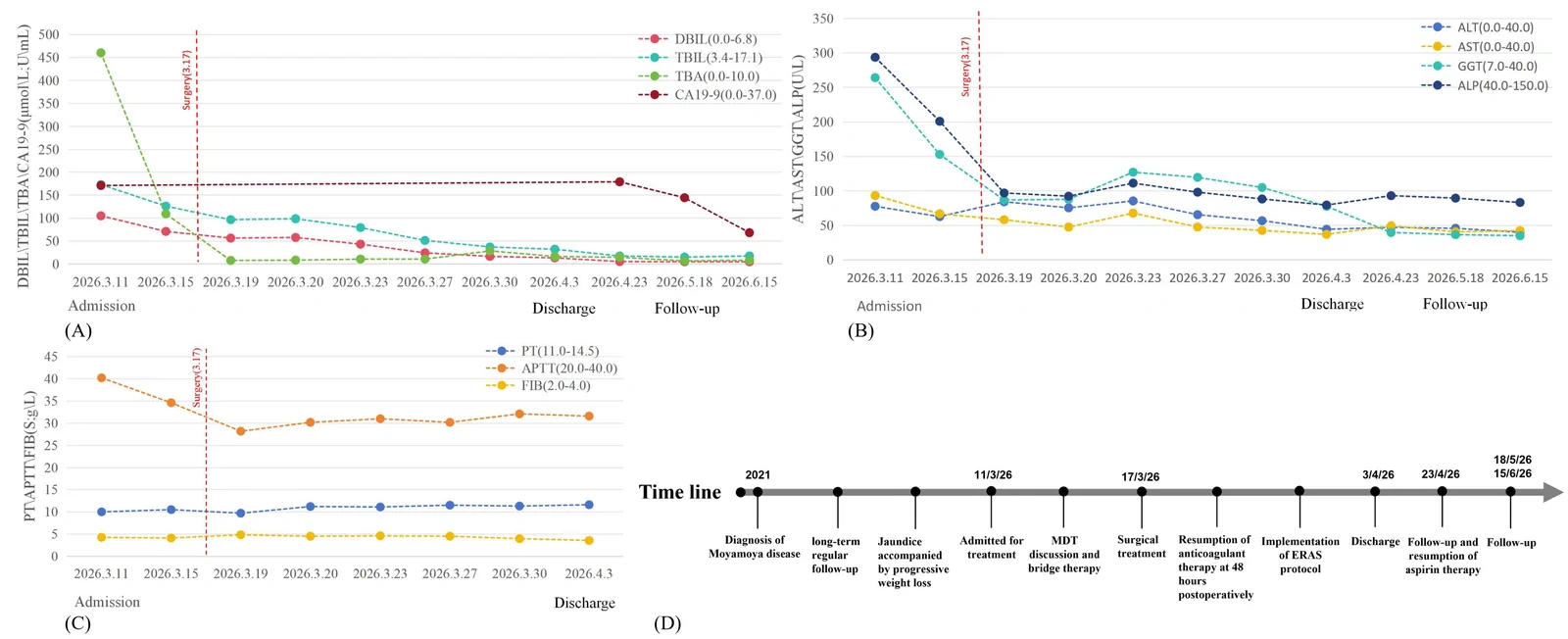
Clinical timeline of patient management and dynamic changes in coagulation parameters, CA19-9, and liver function parameters during the perioperative period and follow-up. **(A, B)** All liver function parameters gradually return to normal ranges, indicating significant improvement in cholestasis and gradual recovery of hepatic function. Postoperative serum CA19–9 levels show a sustained decline, which is an indicator of a favorable prognosis. **(C)** Coagulation-related parameters remain relatively stable preoperatively and postoperatively. **(D)** Shows the flow chart of the patient during treatment. (PT, prothrombin time; APTT, activated partial thromboplastin time; FIB, fibrinogen; ALT, alanine aminotransferase; AST, aspartate aminotransferase; GGT, γ-glutamyl transferase; ALP, alkaline phosphatase; TBIL, total bilirubin; DBIL, direct bilirubin; TBA, total bile acid.) The vertical red dashed line marks the date of surgery (March 17).

The patient had an uneventful postoperative course. Neurological function was assessed daily using the NIHSS, with a score of 0 throughout the perioperative period. No neurological symptoms such as headache, dizziness, limb weakness, speech disturbance or consciousness disturbance were observed. No major surgical complications including grade B/C pancreatic fistula, biliary fistula, intra-abdominal hemorrhage, or surgical site infection (SSI) occurred. Postoperative jaundice resolved gradually, and liver function parameters improved progressively, confirming favorable surgical outcomes (**Figures 3A–C**). The patient recovered well and was discharged uneventfully at 2 weeks postoperatively.

**Figure 4:**
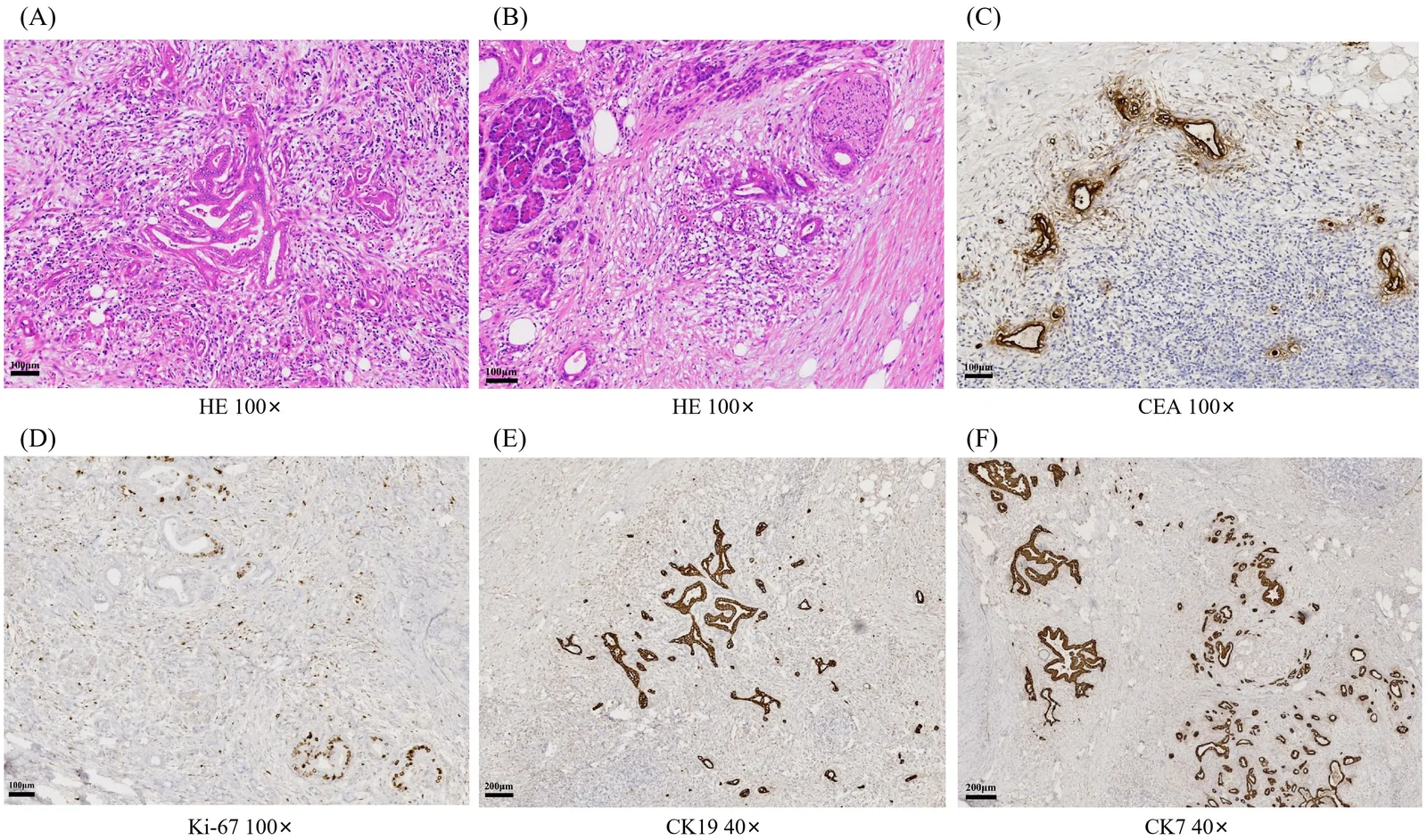
Pathological and immunohistochemical findings of moderately differentiated PDAC. **(A)** Complex atypical glands infiltrate dense desmoplastic stroma with associated chronic inflammatory cell infiltration. **(B)** The tumor infiltrated the pancreatic parenchyma with perineural invasion (PNI). **(C)** Immunohistochemical staining for CEA shows diffuse strong membranous and cytoplasmic positivity in tumor glands, with negative staining in stromal cells. **(D)** Immunohistochemical staining for Ki-67 shows nuclear positivity with a labeling index of approximately 25%, and stromal cells are negative. **(E)** Immunohistochemical staining for CK19 shows diffuse strong positivity, delineating branching infiltrative neoplastic glands. **(F)** Immunohistochemical staining for CK7 shows diffuse strong positivity, clearly delineating irregular infiltrative neoplastic glands. (PDAC, pancreatic ductal adenocarcinoma; HE 100×, Hematoxylin and eosin staining at 100× magnification; CEA 100×, Carcinoembryonic antigen immunohistochemistry at 100× magnification; Ki-67 100×, Ki-67 immunohistochemistry at 40× magnification; CK19 40×, Cytokeratin 19 immunohistochemistry at 40× magnification; CK7 40×, Cytokeratin 7 immunohistochemistry at 40× magnification).

Final histopathological examination confirmed a 1.4 × 1.4 cm tumor located in the pancreatic head. The tumor infiltrated the pancreatic parenchyma with perineural invasion (PNI), and was histologically graded as moderately differentiated pancreatic ductal adenocarcinoma. No metastatic foci were identified in any of the 15 dissected lymph nodes (**Figures 4A, B**). Immunohistochemical staining showed positive expression of cytokeratin 7 (CK7), cytokeratin 19 (CK19), and carcinoembryonic antigen (CEA). The Ki-67 proliferation index was 25% (**Figures 4C–F**). All surgical margins were negative, confirming R0 resection. Based on the AJCC 8th edition criteria, the tumor was classified as pT1cN0M0 stage. Adjuvant chemotherapy with S-1 (60 mg twice daily) was administered based on recommendations from Chinese pancreatic cancer guidelines and the 2022 Japan Pancreas Society (JPS) Clinical Practice Guidelines for Pancreatic Cancer (5, 6). Notably, S-1 is not listed as an adjuvant regimen in the NCCN Guidelines for Pancreatic Adenocarcinoma.

The patient underwent regular follow-up after discharge(Last follow-up date: June 15, 2026). The patient has resumed normal daily activities, with a regular diet, stable neurological function (NIHSS score was 0 at all follow-up time points) and good quality of life, and indicating favorable short-term oncological and neurological outcomes (**Figures 3B, C**). Postoperative serum CA19–9 levels declined sustainably, and imaging examinations indicated no typical signs of recurrence or distant metastasis.

## 3 Discussion

Both MMD and PDAC are rare diseases with low incidence, and their concurrence constitutes an extremely rare clinical scenario that has been scarcely reported in the literature (7, 8). PD is one of the most complex abdominal surgical procedures and demands strict perioperative hemodynamic stability, which is exactly the primary risk factor for cerebrovascular events in patients with MMD (3, 9). The clinical dilemma lies in balancing the need for oncologically radical resection with the prevention of potentially fatal stroke or intracranial hemorrhage.

The clinical challenges include two aspects: (1) intraoperative hemodynamic fluctuations, hypotension, and hypocapnia can directly precipitate cerebral hypoperfusion and ischemic stroke in patients with MMD (10); (2) the high incidence of postoperative pancreatic fistula, hemorrhage, and infection can further exacerbate hemodynamic instability and elevate the risk of cerebrovascular events (11). MDT collaboration is critical for ensuring patient safety, and the strategies are consistent with the most recent high-quality evidence in the field (1, 12, 13).

Patients with MMD have impaired cerebrovascular autoregulation, and cerebral perfusion becomes passively dependent on MAP. Our strategy of maintaining MAP at 80–100 mmHg was designed to ensure adequate cerebral perfusion while avoiding excessive blood pressure that could rupture fragile collateral vessels (1, 12). Continuous rSO_2_ monitoring provided real-time assessment of cerebral oxygenation, allowing immediate intervention for any episodes of hypoperfusion. Maintenance of normocapnia (PaCO_2_ 35–40 mmHg) was critical, as hypocapnia causes cerebral vasoconstriction and can precipitate ischemic stroke in MMD patients, while hypercapnia increases cerebral blood volume and the risk of hemorrhage (4).

Anesthetic management was tailored to minimize hemodynamic perturbations. Etomidate was administered for anesthesia induction to minimize myocardial depression and reduce the risk of severe hypotension during induction. Anesthesia was maintained with sevoflurane combined with remifentanil, which enabled stable regulation of anesthetic depth and facilitated rapid postoperative emergence (14). Shortening operation time and reducing bleeding minimize hemodynamic interference. Furthermore, adhering to standard PD surgical procedures reduces the incidence of postoperative pancreatic fistula (15). ERAS protocols reduce surgical stress and accelerate postoperative recovery, further reducing the risk of cerebrovascular events (16).

Antiplatelet therapy is a cornerstone of primary ischemic prevention for MMD patients. However, the continuous use of aspirin during surgery increases the risk of surgical bleeding, particularly at the pancreatic anastomosis site. Given the substantially elevated perioperative stroke risk in this patient, LMWH was selected as the individualized perioperative bridging anticoagulant in accordance with perioperative antithrombotic management guidelines and therapeutic recommendations from Mayo Clinic practice guidelines. This regimen could effectively reduce the risk of ischemic events during the aspirin withdrawal window, while delayed postoperative anticoagulation resumption was adopted to balance the risk of postoperative pancreatic fistula and intracranial hemorrhage (1, 17, 18).

This report has several inherent limitations. First, it is a single case report and histopathological examination revealed the presence of PNI, a well-established pivotal adverse prognostic factor for pancreatic cancer. Although adjuvant chemotherapy was administered in accordance with consensus clinical guidelines, the follow-up duration remains relatively limited. Long-term follow-up is required to assess both oncological and cerebrovascular outcomes. Second, the management strategy described was developed for a patient with asymptomatic, stable MMD. It may not be directly applicable to patients with symptomatic MMD, a history of cerebrovascular events, or prior cerebral revascularization surgery. Finally, the efficacy of this treatment regimen requires validation through larger multicenter cohort studies.

## 4 Conclusion

The comorbidity of MMD and PDAC represents an extremely rare and high-risk clinical condition. MDT-guided individualized management incorporating three core principles-strict hemodynamic regulation to maintain stable cerebral perfusion, optimized anesthesia and surgical techniques to minimize trauma, and staged balanced anticoagulation to balance bleeding and thrombotic risks-enabled successful radical tumor resection without severe complications. This case provides clinical experience for clinicians encountering similar rare clinical scenarios. However, due to the inherent limitations of the single-case report design, this individualized multidisciplinary strategy is tailored to the present patient, and the efficacy and safety of this strategy remain to be validated in further studies.

## Data Availability

The original contributions presented in the study are included in the article/supplementary material. Further inquiries can be directed to the corresponding authors.

## References

[1] Fujimura M Tominaga T Kuroda S Takahashi JC Endo H Ogasawara K . 2021 Japanese guidelines for the management of moyamoya disease: guidelines from the research committee on moyamoya disease and Japan stroke society. Neurol Med Chir (Tokyo). (2022) 62:165–70. doi: 10.2176/jns-nmc.2021-0382 35197402 PMC9093674

[2] W J L S L R W Y S F P X . Risk factors for perioperative cerebral infarction in moyamoya disease: a meta-analysis. Front Neurol. (2025) 16. doi: 10.3389/fneur.2025.1530137 39926020 PMC11802441

[3] Seo Y Jung H-S Han Y Lee I Choi G-W Chae YS . Long-term oncologic outcomes of robot-assisted pancreaticoduodenectomy versus open pancreaticoduodenectomy for pancreatic cancer. Surg Endosc. (2025) 39:5097–106. doi: 10.1007/s00464-025-11833-y 40634729 PMC12287213

[4] Ogawa S Miyawaki S Imai H Hongo H Umekawa M Kiyofuji S . Cerebrovascular events during treatment for systemic Malignant tumors in patients with moyamoya disease. World Neurosurg. (2023) 179:e314–e320. doi: 10.1016/j.wneu.2023.08.083 37634665

[5] Cui J Jiao F Li Q Wang Z Fu D Liang J . Chinese society of clinical oncology (CSCO): clinical guidelines for the diagnosis and treatment of pancreatic cancer. J Natl Cancer Cent. (2022) 2:205–15. doi: 10.1016/j.jncc.2022.08.006 39036552 PMC11256594

[6] Okusaka T Nakamura M Yoshida M Kitano M Ito Y Mizuno N . Clinical practice guidelines for pancreatic cancer 2022 from the Japan pancreas society: a synopsis. Int J Clin Oncol. (2023) 28:493–511. doi: 10.1007/s10147-023-02317-x 36920680 PMC10066137

[7] Cai J Chen H Lu M Zhang Y Lu B You L . Advances in the epidemiology of pancreatic cancer: Trends, risk factors, screening, and prognosis. Cancer Lett. (2021) 520:1–11. doi: 10.1016/j.canlet.2021.06.027 34216688

[8] Liu Z Wang Y Li C Wang L Zhao Q . Moyamoya disease: epidemiology, clinical characteristics, diagnosis, physiopathology, and treatment. Cerebrovasc Dis. (2025), 1–15. doi: 10.1159/000548746 41166534

[9] Cao X Wang L Ma Z Xi W Li Y Zhang S . Risk factors influencing disease onset and surgical outcomes in moyamoya disease. J Multidiscip Healthc. (2026) 19:561544. doi: 10.2147/JMDH.S561544 41710391 PMC12911982

[10] C Y Z C C X W Y . Inhalational versus intravenous anesthetic for cerebrovascular accident outcomes after surgical revascularization for adult moyamoya disease. BMC Anesthesiol. (2025) 25. doi: 10.1186/s12871-025-02958-7 39955519 PMC11829331

[11] Manoukian G Qureshi MR Bangash A Hussain Y Singh J Navarro G . Management of complications after pancreaticoduodenectomy: a narrative review of pathophysiology and treatment strategies. Cureus. (2026) 18:e101919. doi: 10.7759/cureus.101919 41728445 PMC12917441

[12] Yang KJ Mistry P Ayrian E . Update on the anesthesia management in adult patients with moyamoya disease. Curr Opin Anaesthesiol. (2024) 37:439–45. doi: 10.1097/ACO.0000000000001411 39011661

[13] Li D Lv F Ding C Zhuang Z Wang S . Pregnancy risk assessment, management, and delivery plan for pregnant women with moyamoya disease using a multidisciplinary collaborative approach: a case series. Int J Women’s Health. (2024) 16:1415–24. doi: 10.2147/IJWH.S472646 39221426 PMC11363915

[14] Williams G Jones W Chaudhry R Cai C Pednekar G Long A . Intraoperative anesthesiology management and patient outcomes for surgical revascularization for moyamoya disease: a review and clinical experience. J Neurol Surg A Cent Eur Neurosurg. (2019) 80:143–8. doi: 10.1055/s-0039-1677823 30818408

[15] Azam T Salameen H Wang Y-B Du W-L Woraikat S Wang X-L . Development and validation of a nomogram for predicting postoperative complications after pancreaticoduodenectomy: a retrospective cohort study. Front Oncol. (2026) 16:1839358. doi: 10.3389/fonc.2026.1839358 42245723 PMC13229620

[16] Noba L Rodgers S Doi L Chandler C Hariharan D Yip V . Costs and clinical benefits of enhanced recovery after surgery (ERAS) in pancreaticoduodenectomy: an updated systematic review and meta-analysis. J Cancer Res Clin Oncol. (2023) 149:6639–60. doi: 10.1007/s00432-022-04508-x 36629919 PMC10356629

[17] Douketis JD Spyropoulos AC Murad MH Arcelus JI Dager WE Dunn AS . Perioperative management of antithrombotic therapy: an american college of chest physicians clinical practice guideline executive summary. Chest. (2022) 162:1127–39. doi: 10.1016/j.chest.2022.08.004 35964703

[18] Fujikawa T Naito S . Safety of pancreatic surgery with special reference to antithrombotic therapy: a systematic review of the literature. World J Clin cases. (2021) 9:6747–58. doi: 10.12998/wjcc.v9.i23.6747 34447821 PMC8362514

